# Knowledge, Attitudes and Practices of Cattle Owners Towards Bovine Tuberculosis in and Around Nekemte Town, Western Ethiopia

**DOI:** 10.1002/vms3.70957

**Published:** 2026-04-25

**Authors:** Yihenew Getahun Ambaw, Getacho Aga, Ambachew Motbaynor Wubaye, Simachew Getaneh Endalamew, Alebachew Tilahun Wassie, Simegnew Adugna Kallu

**Affiliations:** ^1^ College of Veterinary Medicine Haramaya University Dire Dawa Ethiopia; ^2^ Department of Veterinary Medicine College of Agricultural Sciences Woldia University Weldiya Ethiopia; ^3^ Department of Veterinary Science College of Agriculture and Environmental Science Debre Tabor University Debre Tabor Ethiopia; ^4^ Department of Veterinary Epidemiology and Public Health School of Veterinary Medicine Bahir Dar University Bahir Dar Ethiopia; ^5^ Department of Veterinary Clinical Medicine School of Veterinary Medicine Bahir Dar University Bahir Dar Ethiopia

**Keywords:** attitude, cattle, knowledge, practices, bovine tuberculosis

## Abstract

**Background:**

Bovine tuberculosis is listed among the top three animal diseases that cause major animal and public health concerns in Ethiopia. Knowledge, attitude and practices (KAPs) of farmers towards bovine tuberculosis in Ethiopia remain insufficiently studied, especially among local cattle owners. Therefore, this study aimed to assess the level of cattle owners’ KAPs.

**Methods:**

A cross‐sectional study with 200 randomly selected study participants was carried out using a face‐to‐face interview from December 2023 to May 2024 in and around Nekemte town, western Ethiopia. The statistical analysis was performed using Stata version 16. Chi‐square analysis was used to assess the association between independent variables and owners' KAPs, and multivariable logistic regression analysis was used to identify potential predictor variables.

**Result:**

Among the total respondents, 34.5% and 32.0% of them knew about bovine tuberculosis and the zoonotic importance of it, respectively, while 18.8% of the respondents knew that the causative agent of bovine tuberculosis is bacteria. The KAPs of the respondents were associated with their education level. Those respondents who attended college/university and secondary education were 5.76 (1.46–22.66) and 3.26 (1.03–12.08) times more knowledgeable about bovine tuberculosis than those who did not attend a formal education. Those respondents who had completed college/university had 7.34 (2.33–23.11), secondary school had 3.34 (1.15–9.66), and primary school had 4.54 (1.48–13.89) times more likely to have a desirable attitude than those who did not attend formal education. With regard to practices, those respondents with a college/university, secondary or primary education had 6.74 (1.97–23.10), 3.53 (1.11–11.27) and 5.13 (1.52–17.32) times more appropriate practices, respectively, than those who did not attend a formal education.

**Conclusion:**

The respondents had a low level of KAPs regarding bovine tuberculosis in and around Nekemte town, Ethiopia. Raising awareness through education and training campaigns, both in human and animal health sectors, using a one health approach, is necessary.

## Graphical abstract

Cattle owners KAPs towards bovine tuberculosis were assessed using a structured questionnaire. Out of 200 study participants 20.5%, 33.5% and 29.0% showed good knowledge, desired attitude and appropriate practices, respectively. Education level was associated with their KAPs, and awareness creation campaigns using a one health approach are necessary.

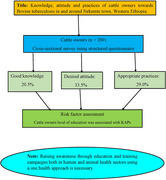

## 1 Introduction

Tuberculosis is one of the globally distributed re‐emerging diseases, resulting in significant economic and mortality loss every year among human beings. The impact of tuberculosis is escalating in less developed countries such as Ethiopia, where there is poor knowledge, attitudes, and practices (KAPs) regarding the disease (Bihon et al. 2021). Since there is no universally approved diagnostic system for bovine tuberculosis (bTB) in humans and animals, the disease has continued to re‐emerge in endemic areas. The communal housing of humans and animals, especially in rural areas, may contribute to the spread of diseases (Sweetline Anne et al. 2017). Bovine tuberculosis is still an ongoing issue in numerous nations, primarily those where test and slaughter regulations cannot be effectively implemented or where domestic animals are frequently infected by wildlife reservoirs of *Mycobacterium bovis* (Buddle et al. 2018).

Worldwide, bTB is a major challenge in the human and animal health sectors, with over 50 million cattle thought to be infected globally and an estimated annual economic burden of approximately US$3 billion (Waters et al. 2012). The disease is a significant zoonosis that can infect humans, especially if they consume unpasteurized milk or live with infected animals. Numerous nations were able to eradicate bTB by introducing the test and slaughter control program, which was implemented in a number of countries in the middle of the 19th century. However, in many poor nations, these control programs have not been financially feasible or socially acceptable, and over 94% of the world's population lives in nations with little to no control over tuberculosis in cattle (Cousins 2001).

Each year, TB kills 1.5 million people and infects 10 million new cases worldwide (World Health Organization (WHO) 2024). Although *Mycobacterium tuberculosis* is the causative agent of tuberculosis in humans, *M. bovis* is the zoonotic pathogen that causes bTB, which is responsible for up to 10% of human infections (World Organisation for Animal Health (OIE) 2024). Bovines are the main reservoir of *M. bovis*, and the inhalation of aerosols and the consumption of dairy products like raw milk and raw meat are the main sources of bTB in humans (Cousins 2001).

Bovine tuberculosis prevention and control requires a multidisciplinary approach that takes into account human, animal and environmental health. The one health approach is being promoted by numerous noticeable organizations as a comprehensive remedy for the problems pertaining to the animal‐human interaction (Zinsstag et al. 2015). For instance, OIE lists bTB as a significant zoonotic and animal disease (World Organisation for Animal Health (OIE) 2014).

Bovine tuberculosis has been identified by the FAO as a significant infectious disease that requires national and regional measures to manage at the animal‐human interface (El Idrissi and Parker 2012). However, bTB still results in significant economic losses by decreasing the productivity and the condemnation of part of or all of the carcasses during slaughter. Because bTB has a detrimental impact on farmers' and nations' economies through livestock deaths, productivity losses from chronic disease and restrictions on animal trade both domestically and internationally, this financial loss has a significant impact on livelihoods, especially in underprivileged and marginalized communities (Cousins 2001).

Extrapolation of information from developed countries and those having low TB burden undermines the percentage of human pulmonary and extra‐pulmonary TB caused by *M. bovis*. Because of this misunderstanding, medical professionals and public health officials are likely unaware of the significance of *M. bovis* as a cause of human TB (Thoen et al. 2010).

Regarding its effects on health and the economy, particularly in low and middle‐income settings, bTB is still a neglected zoonotic disease that receives little attention from research, policy and public health (Weld et al. 2022). In addition, further expenditures increase associated with the surveillance and regular testing of bTB, culling of infected and other in‐contact animals in the herd, and movement restriction on infected herds (Olea‐Popelka et al. 2017).

Studies on KAPs in Ethiopia revealed that most survey participants knew very little about bTB (Bihon et al. 2021, Brennan et al. 2016, Kidane et al. 2015, Asebe and Gudina 2018). Assessing farmers' KAPs is a crucial initial step in developing and implementing disease management and prevention strategies because the community‐based public health education remains the most powerful weapon in promoting awareness among cattle owners (Girma et al. 2023). In Ethiopia, the KAPs of farmers with respect to bTB have not yet been sufficiently examined. Despite the high prevalence of bTB in the study area (Kebede and Kitila 2018, Gemeda et al. 2023), there is insufficient information regarding cattle owners' KAPs. To gain a thorough and accurate understanding of the KAPs of communities, more comprehensive studies on the possible source of infection, the disease's economic and public health burden, its modes of transmission, and its control measures are therefore necessary. Thus, the objective of this study was to quantify cattle owners' KAP levels and identify sociodemographic predictors of good KAPs towards bTB.

## 2 Materials and Methods

### 2.1 Description of Study Area

**FIGURE 1:**
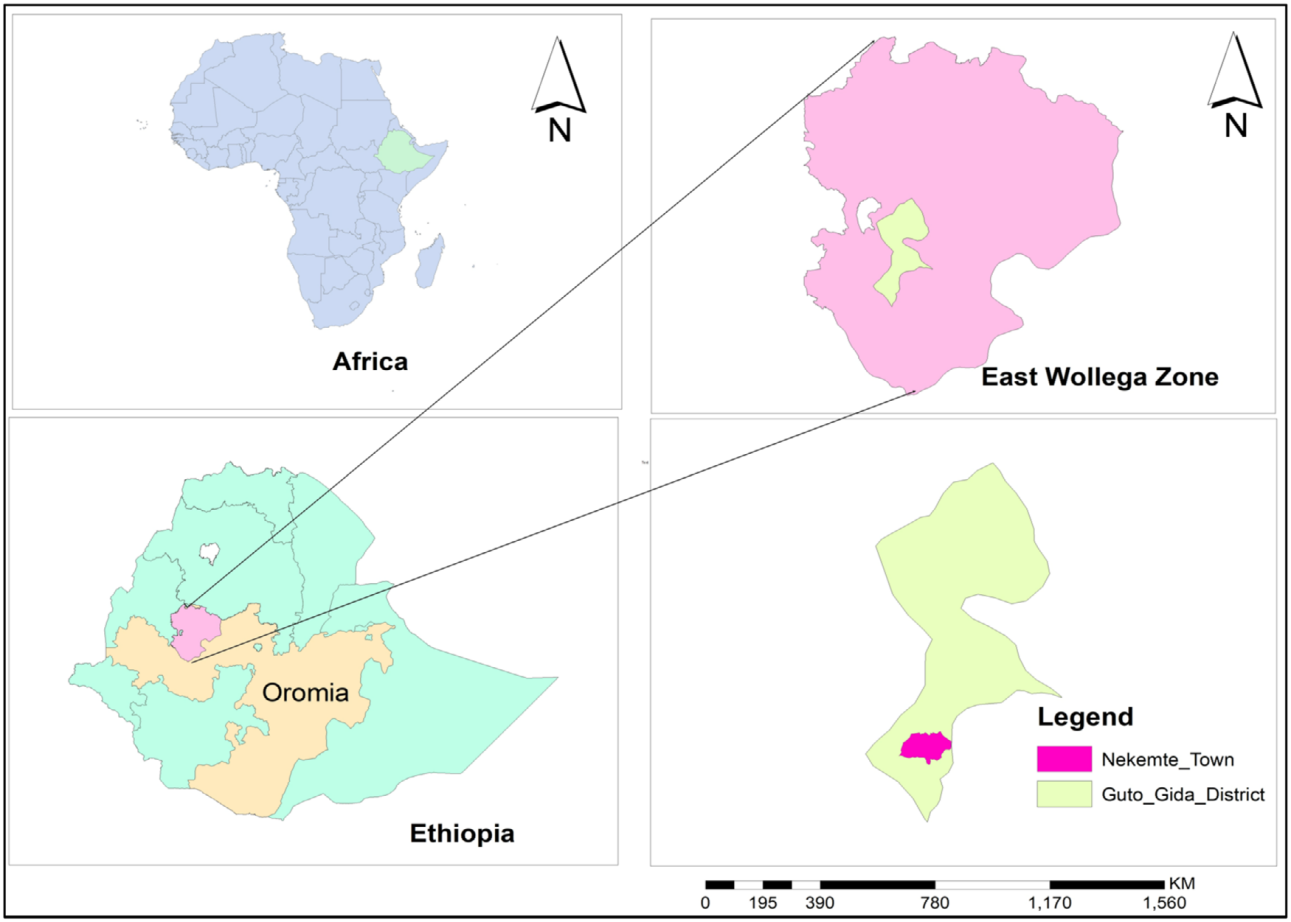
Study area map of Nekemte town and surrounding area.

The study was carried out in and around Nekemte town, which is located in the Guto Gida District, East Wollega Zone, Oromia region, Federal Democratic Republic of Ethiopia (Figure 1). It is situated at a latitude of 09°04′957′′ N, longitude 36°32′928′′ E and elevation of 2124 m above sea level. It is located 331 km from the capital of Ethiopia, Addis Ababa. Rainfall amounts range from 1800 to 2200 mm each year. The area experiences a high temperature of 25°C and a low temperature of roughly 20°C. The region experiences a brief wet season from March to May and a long rainy season from June to September. There is an abundance of native vegetation, which includes all types of grasses, bushes and trees from tropical rainforests. The Guto Gida community raise vast cattle herds and use a mixed farming method, which involves both crop production and animal husbandry. There are 86,724 cattle, 8589 horses, 14,171 sheep, 11,821 goats and 57,695 fowl in the district. The district also has 125,682 people among those 63,760 are male and 61,922 female (Guto Gida District Agriculture Office (GGDAO) 2022).

### 2.2 Study Design

A cross‐sectional study design was used to assess cattle owners' KAPs about bTB between December 2023 and May 2024 in and around Nekemte town, western Ethiopia.

### 2.3 Sample Size Determination

The sample size was calculated using a formula recommended by Bartlett et al. (Bartlett et al. 2001), with the assumption of a 95% confidence interval, 18.6% of previous good KAPs (Bihon et al. 2021), and an absolute error of 5%, which gave an estimated sample size of 233. By considering 10% non‐response rate, the total sample size was calculated to be 256. However, due to the participants' unwillingness, a total of 200 cattle owners consented and responded in the current study, where the response rate was 78.1%.

### 2.4 Sampling Techniques, Data Collection and Measurement Tools

The sampling frame for this survey was obtained from the Guto Gida district agricultural and rural development office, which encompasses the lists of cattle owners. A total of 256 study participants were recruited from those lists by simple random sampling technique using lottery method. During the survey, face‐to‐face interviews were conducted using a structured questionnaire designed to assess the KAPs of cattle owners. To increase the quality of the questionnaire tool, pre‐test was carried out on 25 participants in and around Guder town, which is located near the Nekemte town and minor modification was made and excluded from the final analysis. The internal consistency and reliability of the questionnaire used in this study were assessed using the Cronbach's alpha coefficient, which was 0.78 for knowledge, 0.81 for attitude and 0.73 for practices, indicating internal consistency. In each domain, 20, 9 and 8 items were included, respectively. By using the KAPs tool, information regarding cause and modes of transmission of bTB, owners' habits of consuming raw meat and milk, and the presence of human‐cattle contact were collected.

The participants were given a score of 1 if they responded correctly and 0 if not. Accordingly, a knowledge score ranges from 0–20, an attitude score ranges from 0–9 and the practices score ranges from 0–8. Based on Bloom's theory, the participants were grouped into two categories per domain (good and poor knowledge, desired and undesired attitude and appropriate and inappropriate practices). On the KAP items, respondents who scored more than 50% were considered to have good knowledge, desired attitudes and appropriate practices, while those who scored less than 50% were considered to have poor knowledge, undesired attitudes and inappropriate practices.

### 2.5 Data Management and Analysis

A consistent data extraction format developed in Microsoft Excel version 13 was used to collect the raw data among the respondents, which were then imported into the statistical program Stata version 16 for analysis. The expected association between each predictor variable and KAPs was examined using a chi‐square test. The selection of candidate predictor variables for the multivariable binary logistic regression model was based on *p*‐values less than 0.25. Multicollinearity among independent variables was assessed using variance inflation factor (VIF) analysis. All variables except the main occupation of the participants had VIF values below 5, indicating no multicollinearity. The final multivariable binary logistic regression analysis was conducted for each of the three KAP domains, by incorporating the potential predictor variables simultaneously that had a *p*‐value < 0.25 in the chi‐square analysis. The Hosmer–Lemeshow test was used to assess the final model's goodness of fit. All statistics were considered significant when the *p*‐value was ≤ 0.05.

## 3 Results

### 3.1 Sociodemographic Characteristics of the Study Participants

Among the 200 participants, 127 (63.5%) were male, and more than two‐thirds of the respondents (68.5%) were found in the age category of ≥ 30 years. With respect to educational status, the majority (36.5%) of the respondents attended secondary school. Regarding the participants' occupation, more than one‐third of the respondents (38.0%) were students, and 34.0% were involved in farming activities (Table 1).

**TABLE 1:** Sociodemographic characteristics of the study participants.

Socio‐demographic	Categories	Frequency (%)	95% CI
Residence	Rural	84 (42.0)	35.37–48.93
	Urban	116 (58.0)	51.07–64.63
Gender	Male	127 (63.5)	56.63–69.86
	Female	73 (36.5)	30.14–43.37
Age	18–29	63 (31.5)	25.46–38.23
	≥ 30	137 (68.5)	61.77–74.54
Marital status	Married	102 (51.0)	44.12–57.84
	Unmarried	98 (49.0)	42.16–55.88
Educational level	No formal education	42 (21.0)	15.93–27.16
	Primary school	46 (23.0)	17.71–29.31
	Secondary school	73 (36.5)	30.14–43.37
	College/university	39 (19.5)	14.61–25.54
Main occupation	Farmers	68 (34.0)	27.79–40.81
	Merchant	27 (13.5)	9.45–18.93
	Student	76 (38.0)	31.56–44.89
	Employed	29 (14.5)	10.29–20.05

### 3.2 Knowledge of Cattle Owners About bTB

**FIGURE 2:**
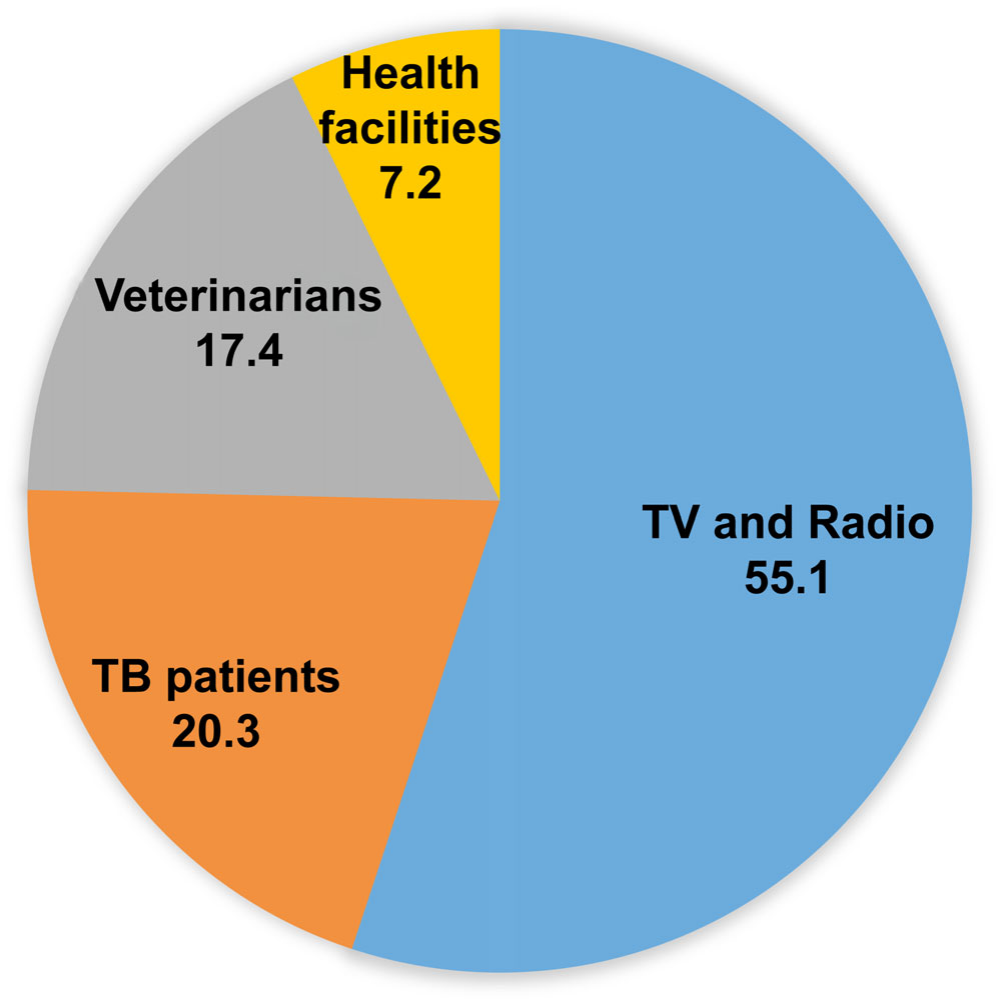
Source of information among respondents for bTB in and around Nekemte town.

When the participants were asked about bTB, 65.5% of them said they had never heard about the disease. Those who heard about bTB were additionally asked about their source of information, and the majority (55.1%) said they got it from television or radio, followed by TB patients (20.3%) (Figure 2).

Among the study participants, one‐third (34.5%) had heard about bTB. From those who heard about bTB, 89.9% and 39.1% of the participants knew that bovine TB transmits from animal to animal and animal to human, respectively, with different transmission mechanisms and 18.5% were aware that the causative agent is bacteria. Among the participants, 62.3% claimed that bTB is a curable disease, and regarding bTB treatment, 81.4%, 11.6% and 7.0% of the participants said that bTB can be cured using specific drug treatment in veterinary clinics, herbal remedy and home rest and pray, respectively. Table 2 summarises knowledge‐related questions with the study participants' responses.

**TABLE 2:** Knowledge related questions about bTB in Nekemte town and participants' response.

Knowledge‐related items	Response, frequency (%)	
	Yes	No
Do you know what bovine TB is?	69 (34.5)	131 (65.5)
Do you know that the causative agent of bovine TB is a bacteria?	13 (18.8)	56 (81.2)
Do you know that bovine TB transmits from animal to animal?	62 (89.9)	7 (10.1)
Do you know that bovine TB transmits from animal to animal through inhalation?	23 (37.1)	39 (62.9)
Do you know that bovine TB transmits from animal to animal through contact?	19 (30.6)	43 (69.4)
Do you know that bovine TB is a curable disease?	43 (62.3)	26 (37.7)
Can herbal remedy cure bovine TB?	5 (11.6)	38 (88.4)
Can specific drug in veterinary clinics cure bovine TB?	35 (81.4)	8 (18.6)
Can home rest and pray cure bovine TB?	3 (7.0)	40 (93.0)
Is bovine TB transmissible from animals to humans?	27 (39.1)	42 (60.9)
Does bovine TB transmit from animal to human through ingestion of raw animal products?	11 (40.7)	16 (59.3)
Does bovine TB transmit from animal to human through inhalation?	5 (18.5)	22 (81.5)
Does bovine TB transmit from animal to human through contact with infected cattle?	4 (14.8)	23 (85.2)
Do you know bovine TB is preventable?	55 (79.7)	14 (20.3)
Does separating the house of animals and humans reduce bovine TB transmission from cattle to human?	6 (22.2)	21 (77.8)
Does consuming or using cooked animal products reduce bovine TB transmission from cattle to human?	11 (40.7)	16 (59.3)
Does keeping the health of animals reduce bovine TB transmission from cattle to human?	12 (44.4)	15 (55.6)
Does covering the mouth and nose (using personal protective equipment) reduce bovine TB transmission from cattle to human?	15 (55.6)	12 (44.4)
Does vaccination of animals reduce bovine TB transmission from cattle to human?	13 (48.1)	14 (51.9)
Does culling of sick animals reduce bovine TB transmission from cattle to human?	3 (11.1)	24 (88.9)

### 3.3 Attitudes of Cattle Owners About bTB

About 15.0% of the respondents believe that bTB only affects poor people, whereas 26.5% of the respondents believe that consuming raw milk was a source of bTB. Less than one‐third of the respondents (28.0%) considered that bTB is a problem in Ethiopia, and 19.5% of the respondents believe that bTB can be effectively prevented by drug treatment. When asked whether bTB can be prevented by educating people about the disease, only 29.0% of the participants agreed; however, the majority of the respondents (90.5%) believed that boiling of milk can prevent milk‐borne diseases (Table 3).

**TABLE 3:** Attitude‐related questions about bTB in Nekemte town and participants' response.

Attitude‐related items	Response, frequency (%)	
	Yes	No
Do you think that bTB can affect only poor people?	30 (15.0)	170 (85.0)
Do you believe that bTB is a zoonotic disease?	63 (31.5)	137 (68.5)
Do you think raw milk can be a source of bTB?	53 (26.5)	147 (73.5)
Do you think raw meat can be a source of bTB?	47 (23.5)	153 (76.5)
Do you believe avoiding common housing with cattle can prevent bTB?	71 (35.5)	129 (64.5)
Do you think bTB is a problem in Ethiopia?	56 (28.0)	144 (72.0)
Do you think bTB is effectively prevented by drug treatment?	39 (19.5)	161 (80.5)
Do you think bTB can be prevented by educating people about the disease?	58 (29.0)	142 (71.0)
Do you think boiling of milk prevent milk borne diseases?	181 (90.5)	19 (9.5)

### 3.4 Practices of Cattle Owners About bTB

Animal owners were asked whether they give advice to TB patients to go to the hospital or not, and approximately 74.0% responded correctly. However, three‐quarter (75.5%) of the respondents mixed their cattle with other persons' cattle. Less than half of the respondents (43.5%) eat raw meat and around two‐thirds (65.5%) of the respondents drink raw milk. When the participants were asked a question, “Is there any contact between cattle and wild animals?” about 44.5% of the respondents said yes. Among the total participants, 28.0% of them share the same house with animals (Table 4).

**TABLE 4:** Practices related questions about bTB in Nekemte town and participants' response.

Practice‐related questions	Response, frequency (%)	
	Yes	No
Do you give advice to TB patients to go to the hospital?	148 (74.0)	52 (26.0)
Does your cattle mix with other persons' cattle?	151 (75.5)	49 (24.5)
Do you go to hospital if you are infected with TB?	195 (97.5)	5 (2.5)
Do you use the same watering point with animals?	4 (2.0)	196 (98.0)
Do you share the same house with animals?	56 (28.0)	144 (72.0)
Is there any contact between cattle and wild animals?	89 (44.5)	111 (55.5)
Do you eat raw meat?	87 (43.5)	113 (56.5)
Do you drink raw milk?	131 (65.5)	69 (34.5)

### 3.5 Overall Knowledge, Attitudes and Practices of Cattle Owners Towards bTB

**FIGURE 3:**
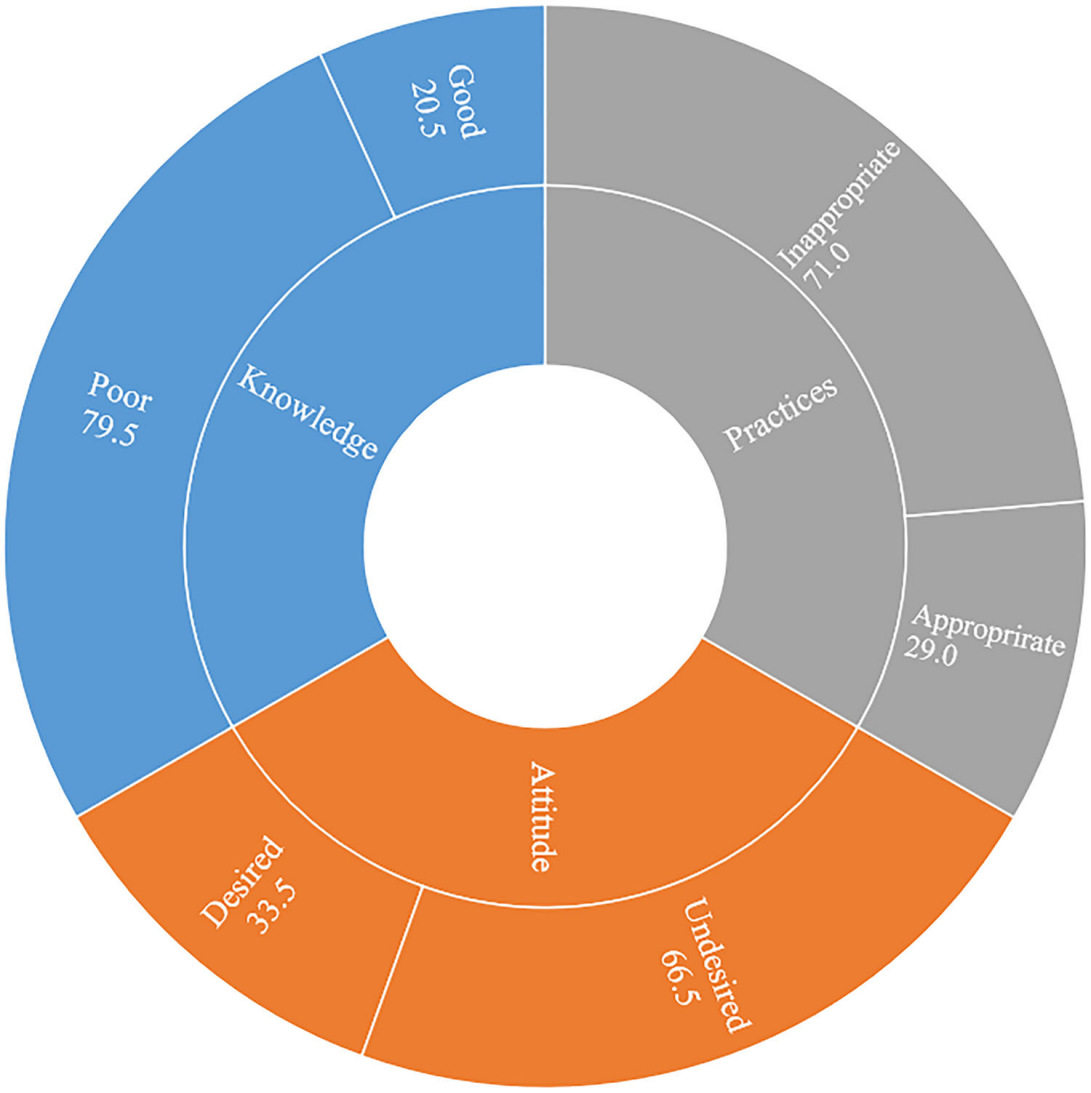
Proportion of respondents' knowledge, attitude and practices regarding bTB in and around Nekemte town.

Among the total respondents, less than one‐fourth (20.5%) had good knowledge, around one‐third (33.5%) had desirable attitude, and less than one‐third (29.0%) had an appropriate practice (Figure 3).

The results of the chi‐square analysis revealed that sex, education level and respondents' occupation were significantly associated with knowledge and practices (*p* < 0.05), but age group, residence and marital status were not (*p* > 0.05). However, only education level and occupation were significantly associated with attitude (*p* < 0.05) (Table 5).

**TABLE 5:** Association of sociodemographic characteristics with respondents' knowledge, attitude and practices towards bTB.

Demographic variables	Categories	Knowledge		*χ* ^2^	*p*‐value	Attitude		χ2	*p*‐value	Practices		χ2	*p*‐value
		Good	Poor			Desirable	Undesirable			Appropriate	Inappropriate		
Sex	Male	14	83	4.25	0.039	28	69	1.82	0.178	21	76	4.94	0.026
	Female	27	76			39	64			37	66		
Age	18–29	12	51	0.12	0.730	20	43	0.13	0.722	15	48	1.20	0.273
	≥ 30	29	108			47	90			43	94		
Residence	Rural	12	72	3.43	0.064	22	62	3.48	0.062	20	64	1.90	0.169
	Urban	29	87			45	71			38	78		
Marital status	Single	22	76	0.45	0.503	35	63	0.42	0.515	27	71	0.20	0.658
	Married	19	83			32	70			31	71		
Education level	No formal education	3	39	10.63	0.014	5	37	16.30	0.001	4	38	13.60	0.003
	Primary school	8	38			17	29			15	31		
	Secondary school	16	57			24	49			21	52		
	College/university	14	25			21	18			18	21		
Main occupation	Farmer	6	62	14.93	0.02	13	55	17.50	0.001	13	55	12.99	0.005
	Merchant	4	23			8	19			7	20		
	Student	19	57			28	48			22	54		
	Civil servant	12	17			18	11			16	13		

*Note: χ* ^2^, chi‐square.

### 3.6 Predictor Factors Associated With Knowledge, Attitudes and Practices of Animal Owners on bTB

Predictor variables included in the multivariable logistic regression analysis based on the chi‐square test were sex, residence and education level. Although main occupation was statistically significant in the chi‐square test, it was excluded from multivariable model due to multicollinearity with education level. According to the multivariable binary logistic regression analysis result, the respondents' education level was statistically associated (*p *< 0.050) with good knowledge. Those respondents who attended college/university and secondary education were 5.78 and 3.26 times more likely to have good knowledge about bTB than those who did not attend a formal education. However, sex and residence of the respondents were not significantly associated with good knowledge (*p* > 0.050) (Table 6). A statistically significant association was found between the respondents' education level and their attitude. Respondents who achieved a college/university, secondary and primary education were 7.34, 3.34 and 4.08, respectively, times more likely to have a desirable attitude about bTB than those who did not attend a formal education (Table 6). The risk factor associated with appropriate practices regarding bTB was education level. When we compared with those who did not attend a formal education, those who completed college/university education were 6.77 times more likely, those who attended secondary school were 3.54 times, and those who attended primary school were 5.73 times more likely to practice appropriately towards the reduction of bTB (Table 6).

**TABLE 6:** Factors associated with good knowledge, desirable attitudes and appropriate practices regarding bTB among animal owners.

Predictor variables	Categories	Good knowledge (*n* = 41)		Desirable attitude (*n* = 67)		Appropriate practice (*n* = 58)	
		aOR (95% CI)	*p*‐value	aOR (95% CI)	*p*‐value	aOR (95% CI)	*p*‐value
Sex	Male	ref					
	Female	1.71 (0.80–3.67)	0.167	1.28 (0.67–2.44)	0.460	1.87 (0.95–3.68)	0.071
Residence	Rural	ref					
	Urban	1.52 (0.70–3.32)	0.294	1.46 (0.76–2.82)	0.257	1.23 (0.62–2.43)	0.559
Educational level	No formal education	ref					
	Primary school	2.99 (0.73–12.28)	0.130	4.54 (1.48–13.89)	0.008^a^	5.13 (1.52–17.32)	0.008^a^
	Secondary school	3.26 (1.03–12.08)	0.049^a^	3.34 (1.15–9.66)	0.026^a^	3.53 (1.11–11.27)	0.033^a^
	College/University	5.76 (1.46–22.66)	0.012^a^	7.34 (2.33–23.11)	0.001^a^	6.74 (1.97–23.10)	0.002^a^
Hosmer–Lemeshow *χ* ^2^ test		2.69	0.847	6.63	0.357	2.65	0.916

*Note*: χ^2^, chi‐square.

Abbreviations: aOR, adjusted odds ratio; CI, confidence interval; *n*, number; ref, reference category.

^a^Statistically significant.

### 3.7 Correlations Between Knowledge, Attitudes and Practices Scores

The bivariate relationships among KAPs scores were evaluated using Spearman correlation coefficient. The pair of each respondents' KAP scales revealed a statistically significant association (p < 0.001). The strongest positive correlation (*ρ* = 0.795) was observed between knowledge and practice scores, followed by attitude and practice scores (*ρ* = 0.644) and attitude and knowledge scores (*ρ* = 0.716) (Table 7).

**TABLE 7:** Spearman correlations between knowledge, attitudes and practices of the study participants in and around Nekemte town.

	Correlations		Knowledge	Attitude
Spearman's rho	Attitude	Correlation coefficient	0.7155	
		*p*‐value	< 0.001	
	Practice	Correlation coefficient	0.7946	0.6436
		*p*‐value	< 0.001	< 0.001

## 4 Discussion

This study was designed to assess KAPs of cattle owners regarding bTB in Nekemte town, western Ethiopia. The level of animal owners' KAPs about specific disease conditions is inexplicably linked to the success of disease control measures. The present study found that only one‐third (34.5%) of the participants had heard of bTB. Similar previous findings were reported from different parts of Ethiopia, including the 35.0% in Adama town (Ameni and Erkihun 2007) and the 31.0% in Lare district (Kidane et al. 2015). However, this result was higher compared to studies conducted in Gondar (24.1%) (Bihon et al. 2021), in Addis Ababa (13.9%) (Brennan et al. 2016), in Mogadishu (19.9%) (Yusuf‐Isleged 2022). However, our finding was lower than the 45.6% in Mana and Limmukosa Districts (Kuma et al. 2013), 55.0% in Central Ethiopia, 91.0% in Ejere town (Asebe and Gudina 2018) and the 98.2% in Senegal (Hamid et al. 2024). Out of those participants who had heard about bTB, only 18.8% of them knew that the causative agent of the disease is bacterial. Although the majority of the participants (89.9%) knew the transmission of bTB from animal to animal, only 37.7% and 30.6% participants understood that the means of transmission of the disease is through inhalation and direct contact with infected animals, respectively. In addition, three‐quarters (75.5%) of the participants use common grazing areas/or watering points with other people and less than half (44.5%) of them also approved that there is contact between their animals with wildlife, which opens close contact between these animals and facilitates the transmission of bTB between cattle and cattle to wildlife. A study conducted between 2006 and 2008 revealed the circulation of bovine TB between cattle and wild animals, which indicated that bTB prevalence was 23% in Ethiopian wildlife (Tschopp et al. 2010).

Among those who are aware of bTB, 39.1% knew that bovine TB can transmit from animals to humans. This result was in agreement with the finding where 32% of the participants knew that cattle could transmit bovine TB to humans (Cousins 2001). However, in this study, only 40.7%, 18.5% and 14.8% of the participants understood that ingestion of animal products, inhalation and contact with infected cattle, respectively, were the modes of transmission of bTB from animal to human. In addition, out of the total participants, 28.0% were sharing house with animals. The close contact between animals and humans in the shared house not only acts as a transmission mode of bTB to humans, but it might also be the cause of human TB transmission to animals, and these animals can be a source of infection to humans with TB (Romha et al. 2018). Although more than three‐quarter (79.7%) of the respondents were aware of that bTB is a preventable disease, less than half of them understood that keeping the health of animals (44.4%), consumption of cooked animal products (40.7%), and keeping cattle in a separate house with human (22.2%) could reduce the transmission of the disease from animal to human. This is a major concern because the owners did not know how they could contract TB from animals, which could lead to not implementing prevention mechanisms. In the current study, less than one‐third of the participants (31.5%) believed that bTB is a zoonotic disease. This is supported by the data, which indicated 2.8% of all human TB cases in Africa are associated with *M. bovis* (World Health Organization (WHO) 2017), which is the most common cause of zoonotic TB and cattle are the major reservoirs.

In this study, about a quarter of the participants believed that raw milk (26.5%) and raw meat (23.5%) consumption could be the source of bTB, and 90.5% of the participants perceived that milk‐borne diseases can be prevented by boiling milk. However, 65.5% and 43.5% of the participants consume raw milk and meat, respectively, which indicates that their perception is not in line with their actions/practices. In addition to the lack of standardized meat inspection practices, the Ethiopian culture of consuming unpasteurized milk and raw meat may increase the transmission of the disease from cattle to humans. This is an indication of taking urgent actions to increase awareness and sensitize cattle owners about the risk of consuming raw/unpasteurized milk and undercooked meat. Behaviour‐change strategies should promote flash‐pasteurization demonstrations using influencers to retain cultural value, build self‐efficacy through women's group boiling training, and amplify cues through routine extension/school messaging, which may improve practices within One Health frameworks.

Of the total participants in this study, 20.5% of cattle owners had good knowledge about bTB, which is comparable with previous reports, such as 24.1% in and around Gondar (Bihon et al. 2021) and 15.0% in Pakistan (Memon et al. 2020). However, the present finding was higher than the 2.5% reported in Senegal (Hamid et al. 2024) and lower than the 37.5% in Nigeria (Hailu et al. 2021). This overall knowledge variation between the participants from different localities could be due to differences in education level, access to information, prevalence of the disease in cattle and humans, and life experiences. For example, the majority of the study participants (37.6%) in Senegal had no education, while in Nigeria, more than half (53.13%) of the respondents had secondary school education and above. Despite most participants citing mass media as an information source, their knowledge remained low. This discrepancy likely stems from the superficial nature of media exposure, where general awareness fails to address specifics like raw milk transmission due to inadequate coverage of zoonotic pathways.

In this study, education level is a predictor variable of the overall knowledge of the respondents. This finding was in agreement with other study findings (Bihon et al. 2021; Abera et al. 2024). Those respondents who had a college/university education, secondary school and primary school had better knowledge about bTB compared to those who did not attend a formal education. The probable justification for this difference could be those respondents who had more education level could have a chance to obtain more information or evidence about the disease in school (Hamid et al. 2024) and the more educated respondents in the community might have a better ability to read and understand regarding to bTB in various sources including social medias, brochures and reports in the concerned institutions. Hence, our study suggests that cattle owners' knowledge could be improved through effective awareness creation campaigns.

The present study also showed that less than one‐third of the participants (29.0%) have appropriate practices regarding bTB. This result was in agreement with other reports from Nigeria (34.3%) (Hailu et al. 2021). Education level of the study participants was the predictor variable on the application of appropriate practices regarding bTB. Accordingly, compared to those who did not have a formal education, respondents who achieved a college/university, secondary and primary education applied appropriate practices regarding bTB mitigation. Similar findings were reported in different study areas of Ethiopia, including in and around Gondar town (Bihon et al. 2021) and Lare district in Gambella region (Kidane et al. 2015). However, a study in Senegal indicated that education level was not significantly associated with respondents' practices about bTB (Hamid et al. 2024).

With respect to the bivariate correlation among the three KAPs domains, there were a strong positive correlation between each domain. Those strong positive correlations disclosed that when one domain increases, the other domain also increases together, or when one domain decreases, the other also decreases. In this study, respondents who had good knowledge of bTB also had desirable attitude and appropriate practices. So, improving one of the KAPs domains will also improve the other KAPs domains.

The low bTB KAP finding in the study area signals One Health gaps addressable via local interventions: extension services for zoonotic risk training, veterinary farm outreach with TB lesion demonstrations and test kits, and school programs promoting safe milk practices. These low‐cost measures through existing infrastructure, prioritized in dairy zones and tracked by repeat KAP surveys, support national policy.

## 5 Conclusion

The study has demonstrated that the overall knowledge, attitudes and practices of cattle owners regarding bTB were low. The level of education was significantly associated with the participants' KAP. Hence, promoting cattle owners' awareness about bTB is critical. To mitigate the impact of zoonotic TB, animal owners are encouraged to implement suggested bTB prevention and control methods such as boiling of milk and well‐cooking of meat before consumption, separating housing for humans and animals, avoiding contact between cattle and wildlife, and seeking veterinary advice for sick animals. Frequent awareness creation and sensitization campaigns using mass media and training programs are crucial to improve cattle owners' KAPs and consequently decrease the impact of bTB on animal and public health.

## 6 Limitations of the Study

Although pretest and pilot study were conducted among 25 respondents around the study area (Gudar town) and attempts were made to ensure that cattle owners appreciated all items properly before they responded, the participants' honesty and recall ability were not autonomously evaluated due to lack of instruments. Chance of social desirability bias where respondents' over or under‐reporting of their KAPs on bTB is also an issue, like other survey studies. Although participants were randomly selected (78.1% response rate), lack of non‐respondent data (21.9%) precludes ruling out systematic differences that could introduce selection bias. Dichotomizing data into binary categories could also hinder the full picture of the participants' KAPs and the study design (cross‐sectional) can affect the cause‐effect relationship of the risk factors and the outcome variables (KAPs) of the respondents. Estimate instability for education levels (wide confidence intervals) limits precision; findings should be interpreted cautiously as hypothesis‐generating.

## Author Contributions

**Yihenew Getahun Ambaw**: conceptualization, methodology, investigation, formal analysis, writing – original draft. **Getacho Aga**: conceptualization, investigation, writing – original draft. **Ambachew Motbaynor Wubaye**: methodology, project administration, supervision, writing – review and editing. **Simachew Getaneh Endalamew**: methodology, supervision, writing – review and editing. **Alebachew Tilahun Wassie**: methodology, validation, writing – review and editing. **Simegnew Adugna Kallu**: methodology, visualization, data curation, formal analysis, writing – review and editing.

## Funding

The authors have nothing to report.

## Ethics Statement

Ethical approval was obtained from Haramaya University, College of Veterinary Medicine Ethical Review Committee (Ref. No: CVM/577/2023). Detailed information was given to cattle owners about the purpose of the study. The study participants were given their consent verbally, and participation was completely voluntarily and they were allowed to leave at any time during the study period.

## Conflicts of Interest

The authors declare no conflicts of interests.

## Data Availability

The data that support the findings of this study are available from the first author (Yihenew Getahun Ambaw) or the corresponding author (Simegnew Adugna Kallu) upon reasonable request.
